# The SNCA-Rep1 Polymorphic Locus: Association with the Risk of Parkinson’s Disease and SNCA Gene Methylation

**DOI:** 10.32607/actanaturae.10956

**Published:** 2020

**Authors:** E. V. Iakovenko, N. Yu. Abramycheva, E. Yu. Fedotova, S. N. Illarioshkin

**Affiliations:** Research Center on Neurology, Moscow, 125367 Russia

**Keywords:** Parkinson’s disease, DNAs methylation, alfa-synuclein gene, SNCA-Rep1

## Abstract

Neurodegeneration in Parkinson’s disease is characterized by the accumulation of alpha-synuclein, a protein encoded by the *SNCA *gene, in neurons. In addition to mutations, many polymorphisms have been identified in this gene, and one of these is a dinucleotide microsatellite: *SNCA*-Rep1*. *The mechanisms by which specific configurations of *SNCA-*Rep1 may contribute to the development of this disease have yet to be clarified. In our study, a relationship between long *SNCA-*Rep1 alleles and Parkinson’s was confirmed in the Russian population. Long allelic variants of *SNCA*-Rep1 were shown to be associated with the hypomethylation of the CpG-sites in intron 1 of the *SNCA *gene. Long variants of *SNCA*-Rep1 are supposed to exert their effect through the hypomethylation of a transcriptionally significant region of this gene. Hypomethylation is usually associated with increased expression, which, in turn, contributes to alpha-synuclein accumulation in neuronal cytoplasm, with the latter being the main molecular marker of Parkinson’s disease. Further studies are needed to establish a relationship between our finding and *SNCA *gene expression.

## INTRODUCTION

Parkinson’s disease (PD) is the second most common neurodegenerative disease after Alzheimer’s, affecting 2% of subjects over 60 years of age [1]. PD is accompanied by a degeneration of the dopaminergic neurons of the substantia nigra, aggregation of alpha-synuclein in the neurons, and the formation of intracellular inclusions (Lewy bodies) [2]. The clinical picture of PD is a combination of bradykinesia with rigidity, rest tremor, postural instability, and a wide range of non-motor symptoms (mental, autonomic, sensory, cognitive, etc.) [3, 4].

Based on its etiology, PD may be classified into two groups: hereditary and sporadic. The hereditary forms of PD are characterized by the presence of a genetically determined pathology (gene mutations) associated with the development of the disease. To date, more than 20 causal PD genes have been identified, the main ones being *SNCA*, *PARK2 *(*Parkin*), *LRRK2*, and *GBA *[5]. However, monogenic forms with various inheritance types account for only about 10–20% of PD cases [6]. The other cases are multifactorial polygenic forms whose pathogenesis is associated with both a genetic component in the form of a predisposition to neurodegeneration and various exogenous factors (intoxication with herbicides, traumatic brain injury, exposure to heavy metals, nutritional features, level of air pollution), as well as physiological aging [7].

To date, genome-wide association studies (GWAS) have identified many genes associated with PD predisposition [8-10]. These studies have revealed risk loci both in genes that have not been considered to be associated with the pathogenesis of PD and in genes that have been known to be associated with a predisposition to the disease. Among these, of considerable interest is the *SNCA *gene located on chromosome 4, which encodes the alpha-synuclein protein. The Ala53Thr missense mutation in the *SNCA *gene, which was described in 1997 in an Italian family with an autosomal dominant type of inheritance, was the first mutation identified in hereditary forms of PD [11]. The *SNCA* gene mutations include both duplications and triplications of certain gene regions, as well as point mutations [12, 13]. A pathomorphological study of brain regions in patients with *SNCA *multiplications revealed an increased alpha-synuclein expression, which may indicate a direct relationship between the dosage of the gene and its expression level [14, 15]. Overexpression of alpha-synuclein is also encountered in the brain samples of sporadic PD patients. Therefore, these findings indicate the universal role of increased alpha-synuclein expression in the pathogenesis of most PD forms [16].

In addition to *SNCA *mutations that lead to hereditary forms of PD, a lot of polymorphisms which increase susceptibility to the sporadic form of PD have been identified in the alpha-synuclein gene. These include a number of single nucleotide polymorphisms located mainly at the 3’-end of the *SNCA *gene, as well as the *SNCA*-Rep1 polymorphic locus in the 5’ region of *SNCA *[17]. The *SNCA*-Rep1 locus is located approximately 10 kbp upstream of the transcription start site and is a polymorphic microsatellite with a different number of dinucleotide repeats. This polymorphism, consisting of dinucleotide repeats (TC)x(TT)1(TC) y(TA)z(CA)w, the number of which varies from 8 to 13, was first described by Xia et al. in 1996 [18]. Six alleles of the *SNCA *gene are distinguishable depending on the number of their repeats. Short alleles (with a smaller number of repeats) are considered protective, while longer alleles can increase the risk of PD [19, 20]. However, despite the progress achieved in indentifying the polymorphisms associated with a predisposition to PD, the mechanisms that underwrite their influence on the development of the disease have remained unexplored.

In recent years, an important role in the development of various multifactorial diseases (mainly cancers, which are the most studied diseases) was assigned to the epigenetic mechanisms that regulate the level of gene expression in various body systems without changing the nucleotide DNA sequence. These main epigenetic mechanisms include gene methylation, histone modifications, and the expression of non-coding RNAs [21]. The epigenome is intimately associated with environmental factors; in contrast to the genome, it can change during life under the influence of lifestyle changes, nutrition, and concomitant pathology [22, 23].

The epigenetic mechanisms associated with the pathogenesis of PD have only relatively recently begun to be studied. These studies have focused mainly on DNA methylation, which is a process of methyl group attachment to cytosine in a CpG dinucleotide (CpG site). CpG sites often accumulate in the transcriptionally significant regions of the gene (promoter and regulatory regions), forming the so-called CpG islands (CpG-rich regions). CpG islands occur in the promoter regions of 60% of the protein-coding genes. CpG islands are predominantly unmethylated, while up to 70–80% of all CpG sites are methylated at the full genome level. Hypermethylation of CpG sites was found to disrupt the binding of DNA polymerases and the transcription factors to these regions, inhibiting gene expression; conversely, hypomethylation is usually associated with increased transcription of the appropriate mRNA and enhanced gene expression [21]. CpG islands are located in the promoter region and intron 1 of the *SNCA* gene. Given that exon 1 of the alpha-synuclein gene is non-transcribed, and transcription begins with exon 2, located immediately after intron 1, it was suggested that the methylation level of these regions may affect transcription, the alpha-synuclein expression level and, possibly, the risk of developing the sporadic form of PD [24].

We studied the effect of the *SNCA*-Rep1 polymorphic locus length on the risk of developing PD, as well as on the methylation level of the CpG sites in the* SNCA *gene.

## EXPERIMENTAL

Our study of the association between the *SNCA*-Rep1 polymorphic locus length and the risk of developing the PD included 460 patients (212 males and 248 females). The mean age of the patients was 55.1 ± 13.5 years; the mean age at the disease onset was 49.9 ± 12.5 years, the mean disease duration was 5.5 ± 4.3 years; all patients were at Hoehn-Yahr stage II or III of PD (mean, 2.3 ± 0.4). All the patients included in the study received antiparkinsonian therapy (usually, two or more drugs); in this case, more than 80% of the patients received dopaminergic therapy (dopamine receptor agonists and/or levodopa drugs). Parkinson’s was diagnosed based on the International Parkinson and Movement Disorder Society criteria. The control group consisted of 460 healthy individuals of comparable gender and age.

The genomic DNA was isolated from peripheral blood leukocytes using a Wizard Genomic DNA Purification kit (Promega, USA). Genotyping of dinucleotide repeats was performed by fragment analysis on an ABI Prism 3130 capillary genetic analyzer (Applied Biosystems/HITACHI). DNA fragments containing tandem repeats were amplified using forward (5’- (Fam)CCTGGCATATTTGATTGCAA-3’) and reverse (5’-GACTGGCCCAAGATTAACCA-3’) primers. The data were processed using the GeneMapper software v. 4.0 (Applied Biosystems).

The study of the association between a *SNCA*-Rep1 polymorphic locus length and the methylation level of the promoter and intron 1 of the *SNCA *gene included 44 PD patients randomly selected from the main group (18 males, 26 females; mean age, 58.2 ± 9.7 years; age of the disease onset, 50.5 ± 10.9 years) and 20 healthy individuals of comparable gender and age (control group).

We analyzed the methylation level of six CpG sites in the *SNCA *gene promoter region (CpG sites from 4–9, numbering from the promoter start site) and 22 CpG sites at the 3’-end of intron 1 (CpG sites from 27–8, numbering from the intron 1 start site). The methylation pattern was determined by direct sequencing of appropriate DNA regions after bisulfite conversion using an EZ DNA Methylation kit (Zymo Research, USA). The results were visualized using the Sequencing Analysis software v.5.2 (Applied Biosystems). The methylation level was calculated by analyzing the primary results of Sanger sequencing. In this case, the percentage of methylation of each specific CpG site in each DNA sample was calculated based on a ratio of the peak C height (an electrophoregram peak whose position corresponds to the analyzed CpG site, indicating the presence of methylated cytosine) to the total height of C+T peaks for a given position (methylated and unmethylated cytosine).

Statistical analysis was performed using the Statistica 10 software (Statsoft Russia). Allelic variants were compared using the chi-square test (multidimensional contingency table); the Bonferroni correction was used for multiple comparisons. The relationship between the polymorphism length and the methylation level was assessed using the multiple linear regression. The critical significance level was set to 0.05.

## RESULTS

We studied a polymorphic microsatellite, *SNCA*-Rep1, located in the alpha-synuclein gene promoter region. Genotype matrices of the PD group and control group are presented in *Table 1*. Fragment analysis showed that Rep1-261 was the most common allele in the PD group and the control group (66.4 and 66.2%, respectively).

**Table 1:** Genotype matrix in the PD group and control group for allelic variants of the SNCA-Rep1 polymorphism

PD group (n = 460)							Control group (n = 460)					
	255	257	259	261	263	265	255	257	259	261	263	265
255	0						0					
257	0	0					0	0				
259	0	0	37				0	0	36			
261	0	2	148	209			1	2	184	193		
263	0	0	18	41	3		0	0	8	33	0	
265	0	0	0	2	0	0	0	0	0	3	0	0

A conditional classification of allelic variants into short (*SNCA*-Rep1-255, -257, and -259), intermediate (*SNCA*-Rep1-261), and long (*SNCA*-Rep1-263 and -265) demonstrated that long alleles were more common in PD than short ones. The allele frequency distribution in subgroups is presented in *Table 2*.

**Table 2:** Occurrence frequency of subgroups of SNCA-Rep1 polymorphism alleles

Site	β-coefficient		P
	first SNCA-Rep1 allele	second SNCA-Rep1 allele	
1. Short: SNCA-Rep1-255, -257, -259	242 (26.3%)	267 (29.0%)	p_1,2,3_ = 0.049^*^ p_1,2_ = 0.34 p_2,3_ = 0.038 p_1,3_ = 0.014^#^
2. Intermediate: SNCA-Rep1-261	611 (66.4%)	609 (66.2%)	
3. Long: SNCA-Rep1-263, -265	67 (7.3%)	44 (4.8%)	

Note. The results are presented as the absolute number of alleles; in parentheses, the percentage of the total number in a group; ^*^ p < 0.05; ^#^ – significant with a Bonferroni correction.

We determined the association between a* SNCA*-Rep1 polymorphic locus length and the methylation level of the promoter region and intron 1 of the* SNCA *gene. The relationship between the methylation level of an individual CpG site and the length of both allelic *SNCA*-Rep1 polymorphisms was analyzed in each patient (the first allele is shorter; the second is longer or equal in length).

No association between the methylation level of the CpG sites of the promoter region and the *SNCA*-Rep1 length was found either in the PD group or in the control group.

In the PD group, four closely spaced CpG sites were located in the intron 1 region of the *SNCA *gene; in this case, a longer *SNCA*-Rep1 polymorphism correlated with a hypomethylation of these sites. The results are presented in *Table 3*. In the controls, there were no statistically significant associations between the studied parameters.

**Table 3:** Association between methylation of CpG sites and the SNCA-Rep1 polymorphic allele length in PD patients

Site	β-coefficient		P
	first SNCA-Rep1 allele	second SNCA-Rep1 allele	
CpG-38	–0.523^***^	–0.164	0.00004^#^
CpG-41	–0.463^**^	–0.202	0.0001^#^
CpG-42	–0.384^*^	–0.331^*^	0.00005^#^
CpG-44	–0.542^***^	–0.163	0.00002^#^

Note. The first SNCA-Rep1 allele is a shorter length allele, the second SNCA-Rep1 allele is a longer or equal-length allele; ^*^ p < 0.05, ^**^ p < 0.01; ^***^ p < 0.001; ^#^ – significant with a Bonferroni correction.

It should also be noted that the PD group included a heterozygous carrier of the rare “long” allele* SNCA*-Rep1-265. This patient had a low methylation level of the CpG sites compared to other patients. In the PD group, the mean methylation level in the intron region was 15.8 ± 5.4% for all CpG sites, while the mean methylation level in the carrier of the “long” *SNCA*Rep1- 265 allele was 10.7%. The highest methylation level (33.9%) was observed in one of the homozygous carriers of the “short” allele *SNCA*-Rep1-259.

## DISCUSSION

In our study, an association between the *SNCA*-Rep1 polymorphic allele and the risk of PD was revealed. The association between long *SNCA*-Rep1 alleles and an increased risk of PD has been demonstrated in a number of studies, while shorter alleles are associated with a reduced risk of developing the disease [19, 20]. In our sample, a protective effect of short alleles could not be confirmed, while long *SNCA*-Rep1 alleles were significantly associated with the development of PD. In addition, patients with the long *SNCA*-Rep1-263 allele showed a predisposion to an earlier onset of the disease; on the contrary, in patients with the short* SNCA*-Rep1-259 allele, the disease tended to begin at a later age [25], which also confirms the predisposing effect of long alleles and the protective effect of short ones.

The impact of the length of *SNCA*-Rep1 on the risk of developing PD is associated with changes in gene expression. Several studies have established an increase in the *SNCA *expression in peripheral blood and in the central nervous system in PD. In this case, the expression level of the *SNCA *mRNA and alphasynuclein protein depends on the length of the *SNCA*Rep1 polymorphism: a larger number of dinucleotide polymorphism repeats is associated with higher gene expression [26-30]. The explanation of the fact that long *SNCA*-Rep1 alleles predispose one to PD via increased* SNCA *expression may be interlinked to both the polymorphism that changes the binding of transcription factors and epigenetic modifications: e.g., in the methylation of the regulatory regions of the *SNCA* gene, which, in turn, may also directly affect transcription.

The results of investigations of the alpha-synuclein gene methylation level in PD patients are inconclusive: some studies have revealed significant *SNCA *hypomethylation in PD compared to the controls [24, 30-34], while others have found no difference between the groups [35-37]. The methylation level has been studied both in brain cells (substantia nigra pars compacta, frontal cortex, cerebellum) and in peripheral blood leukocytes. In this case, the level of methylation in the central nervous system has been comparable to that in the peripheral blood: so, the methylation pattern in blood leukocytes may be used as an analogue of methylation in the brain [34, 38].

The association between the methylation level and various genetic polymorphisms has also been studied. Some single nucleotide polymorphisms have been found to be associated with *SNCA *hypomethylation in PD patients [39, 40]. A correlation between the *SNCA* methylation level and the *SNCA*-Rep1 polymorphic locus length has also been revealed: *SNCA *intron 1 hypomethylation was found to be more pronounced in* SNCA*-Rep1-263 genotype carriers than in short allele carriers [30].

In our study, we assessed the effect of the *SNCA*Rep1 polymorphic locus length on the methylation level of various CpG sites in the promoter region and intron 1 of the *SNCA *gene. We did not find differences in the promoter region methylation level. However, there was a clear association between the methylation level of* SNCA *gene intron 1 and the *SNCA*-Rep1 length. In the PD group, a shorter *SNCA*-Rep1 allele length was associated with a higher methylation level of four closely spaced CpG sites, and, conversely, a longer length was associated with a lower methylation level. Our findings are consistent with the results of earlier studies; in this case, other studies have also revealed a difference in the methylation level in *SNCA *intron 1, which may be an indication of its high transcriptional significance [30]. Therefore, hypomethylation of this region is typical of long *SNCA*-Rep1 alleles, predisposing to the disease.

Given the variability of epigenetic phenomena, the* SNCA *methylation pattern appears to be secondary to *SNCA*-Rep1 genetic variants; however, further research is needed to confirm this suggestion. At present, most researchers think that epigenetic phenomena underlie the influence of many polymorphisms in the noncoding regions associated with multifactorial diseases.

## CONCLUSION

In this study, we analyzed the association between the *SNCA*-Rep1 polymorphic locus length and the risk of developing PD and the methylation level of the promoter and intron regions of the *SNCA *gene–a key factor of this disease. The obtained results confirm an association between carriage of long *SNCA*-Rep1 alleles and an increased risk of developing PD, as well as the relationship between long *SNCA*-Rep1 alleles and hypomethylation of *SNCA *gene intron 1. Therefore, the relationship between the phenotype, genotype, and epigenotype was revealed in the PD–long *SNCA*-Rep1 alleles–*SNCA *hypomethylation series. Further studies are needed to analyze the contribution of epigenetic modifications to the molecular pathogenesis of the disease, which would facilitate the development of fundamentally new technologies for epigenetic correction, which seems to be one of the most promising areas of targeted therapy for the neurodegenerative process.

## Abbreviations

PD: Parkinson’s disease
mRNA: messenger RNA
CpG: cytosine–guanine dinucleotide
SNCA: alfa-synuclein gene
